# The influence of allosteric modulators and transmembrane mutations on desensitisation and activation of α7 nicotinic acetylcholine receptors

**DOI:** 10.1016/j.neuropharm.2015.05.006

**Published:** 2015-10

**Authors:** Anna Chatzidaki, Jarryl M. D'Oyley, JasKiran K. Gill-Thind, Tom D. Sheppard, Neil S. Millar

**Affiliations:** a Department of Neuroscience, Physiology & Pharmacology, University College London, London, United Kingdom; b Department of Chemistry, University College London, London, United Kingdom

**Keywords:** Nicotinic acetylcholine receptor, Ion channel, Allosteric modulation, Pharmacology, 4BP-TQS, *cis*-*cis*-4-(4-bromophenyl)-3*a*,4,5,9*b*-tetrahydro-3*H*-cyclopenta[*c*]quinoline-8-sulfonamide, MLA, methyllycaconitine, nAChR, nicotinic acetylcholine receptor, NS-1738, 1-(5-chloro-2-hydroxy-phenyl)-3-(2-chloro-5-trifluoromethyl-phenyl)-urea, PAM, positive allosteric modulator, TBS-345, 4-(3-(4-bromophenyl)-5-phenyl-1*H*-1,2,4-triazol-1-yl)benzenesulfonamide, TBS-346, 4-(3-(4-bromophenyl)-5-(4-methoxyphenyl)-1*H*-1,2,4-triazol-1-yl)benzenesulfonamide, TBS-516, 4-(5-benzyl-3-(4-bromophenyl)-1*H*-1,2,4-triazol-1-yl)benzenesulfonamide, TBS-546, 4-(3-(4-bromophenyl)-5-propyl-1*H*-1,2,4-triazol-1-yl)benzenesulfonamide, TBS-556, 4-(3-(4-bromophenyl)-5-phenethyl-1*H*-1,2,4-triazol-1-yl)benzenesulfonamide, TQS, *cis*-*cis*-4-(napthalen-1-yl)-3*a*,4,5,9*b*-tetrahydro-3*H*-cyclopenta[*c*]quinoline-8-sulfonamide

## Abstract

Acetylcholine activates nicotinic acetylcholine receptors (nAChRs) by binding at an extracellular orthosteric site. Previous studies have described several positive allosteric modulators (PAMs) that are selective for homomeric α7 nAChRs. These include type I PAMs, which exert little or no effect on the rate of receptor desensitisation, and type II PAMs, which cause a dramatic loss of agonist-induced desensitisation. Here we report evidence that transmembrane mutations in α7 nAChRs have diverse effects on receptor activation and desensitisation by allosteric ligands. It has been reported previously that the L247T mutation, located toward the middle of the second transmembrane domain (at the 9′ position), confers reduced levels of desensitisation. In contrast, the M260L mutation, located higher up in the TM2 domain (at the 22′ position), does not show any difference in desensitisation compared to wild-type receptors. We have found that in receptors containing the L247T mutation, both type I PAMs and type II PAMs are converted into non-desensitising agonists. In contrast, in receptors containing the M260L mutation, this effect is seen only with type II PAMs. These findings, indicating that the M260L mutation has a selective effect on type II PAMs, have been confirmed both with previously described PAMs and also with a series of novel α7-selective PAMs. The novel PAMs examined in this study have close chemical similarity but diverse pharmacological properties. For example, they include compounds displaying effects on receptor desensitisation that are typical of classical type I and type II PAMs but, in addition, they include compounds with intermediate properties.

## Highlights

• A series of novel positive allosteric modulators (PAMs) is described.
• The series of PAMs display differing effects on α7 nAChR desensitisation.
• Transmembrane mutations influencing PAM activity are examined.
• Transmembrane mutations can convert PAMs into agonists.
• Identification of a mutation with differing effects on type I and type II PAMs.

## Graphical abstract

**Figure:**
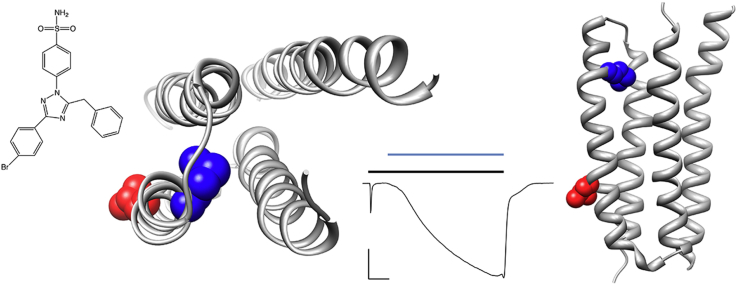

## 1 Introduction

Nicotinic acetylcholine receptors (nAChRs) are transmembrane receptors that are activated by the neurotransmitter acetylcholine. They are members of a structurally related family of pentameric ligand-gated ion channels that also includes receptors for neurotransmitters such as 5-hydroxytryptamine (5-HT), γ-aminobutyric acid (GABA), and glycine (Changeux, 2012; Lester et al., 2004). In common with other members of this family, the conventional ‘orthosteric’ agonist-binding site of nAChRs is located at an extracellular location at the interface of two adjacent subunits (Arias, 2000; Unwin, 2005).

Nicotinic receptors are present at the neuromuscular junction where they mediate muscle contraction (muscle-type nAChRs) and also within the central and peripheral nervous system (neuronal nAChRs), where they have a variety of roles, including mediating fast-synaptic transmission and regulating the release of neurotransmitters (Albuquerque et al., 2009; Gotti et al., 2009). Seventeen subunits have been identified in vertebrates (α1-10, β1-4, γ, δ and ε), which co-assemble to form a diverse family of receptor subtypes (Millar and Gotti, 2009). Whereas most nAChRs are heteromeric combinations of more than one type of subunit (Millar and Gotti, 2009), some nAChRs, such as α7, are able to form functional homomeric nAChRs (Couturier et al., 1990). Homomeric α7 nAChRs are somewhat atypical in that they undergo very rapid desensitisation in response to agonist activation. Indeed, with high concentrations of acetylcholine, almost complete inactivation of α7 nAChRs is observed within milliseconds of agonist activation (Couturier et al., 1990). However, rapid desensitisation of α7 nAChRs is not seen with all agonists. A group of compounds, described as allosteric agonists, activate α7 nAChRs with minimal levels of desensitisation (Gill et al., 2012, 2011; Papke et al., 2014). In contrast to conventional orthosteric agonists such as acetylcholine, there is evidence that allosteric agonists may act via a distinct transmembrane binding site (Gill et al., 2011).

Previous studies have identified an extensive series of compounds that lack agonist activity on α7 nAChRs but which potentiate agonist-evoked responses (Faghih et al., 2008; Williams et al., 2011). Such compounds are described as positive allosteric modulators (PAMs) and, in the case of those acting on α7 nAChRs, have been referred to as either ‘type I’ or ‘type II’ PAMs, depending on their effects on receptor desensitisation (Bertrand and Gopalakrishnan, 2007). The convention is to describe PAMs with little or no significant effect on desensitisation as type I PAMs and those causing a dramatic reduction in desensitisation as type II PAMs. In addition, there is evidence that relatively small changes in chemical structure of allosteric modulators acting on α7 nAChRs can have a dramatic effect on pharmacological properties. For example, altering methyl substitution on a single aromatic ring, which can also alter stereochemistry, has been reported to convert PAMs into negative allosteric modulators, silent allosteric modulators or allosteric agonists (Gill-Thind et al., 2015).

Neuronal nAChRs have been implicated in a variety of cognitive and neurological disorders, including Alzheimer's disease, Parkinson's disease, epilepsy and schizophrenia (Changeux and Taly, 2008; Weiland et al., 2000) and, as a consequence, are targets for therapeutic drug development. A number of subtype-selective orthosteric agonists, partial agonists and antagonists have been developed (Gündisch and Eibl, 2011), but it has been argued that compounds binding to allosteric sites may provide an opportunity for greater receptor subtype selectivity (Williams et al., 2011). Indeed promising results have been obtained in pre-clinical studies with nAChR allosteric modulators in studies examining effects on cognitive deficits (Hurst et al., 2005; Ng et al., 2007; Timmermann et al., 2007), nociception (Munro et al., 2012; Zhu et al., 2011) cerebral ischaemia (Kalappa et al., 2013) and depression (Targowska-Duda et al., 2014).

Here, we describe studies with α7 nAChRs containing one of two point mutations in the second transmembrane domain (TM2) and we examine the effect of these mutations on receptor activation and desensitisation. Introduction of a single point mutation (L247T) in the 9′ position of TM2 of the α7 nAChR has been reported previously to exert dramatic and diverse effects on the functional properties of this receptor (Bertrand et al., 1992; Revah et al., 1991). The effects of the L247T mutation include increased potency of agonists such as acetylcholine and reduced levels of desensitisation (Bertrand et al., 1992; Revah et al., 1991). In contrast, the M260L mutation, located higher up in TM2 (at the 22′ position), does not result in any difference in receptor desensitisation compared to wild-type receptors. Here, we have investigated the effects of these mutations on receptor activation using a variety of PAMs, including a series of novel compounds containing a substituted triazole group. We conclude that multiple transmembrane mutations in α7 nAChRs can convert PAMs into allosteric agonists. In addition, it appears that the M260L mutation has a selective effect on PAMs that reduce agonist-evoked desensitisation (type II PAMs).

## 2 Materials and methods

### 2.1 Chemical synthesis

As is described in more detail in the Supporting information, the following compounds were prepared by a modification of a literature procedure for triazole synthesis (El Kaim et al., 2010): 4-(3-(4-bromophenyl)-5-phenyl-1*H*-1,2,4-triazol-1-yl)benzenesulfonamide (TBS-345), 4-(3-(4-bromophenyl)-5-(4-methoxyphenyl)-1*H*-1,2,4-triazol-1-yl)benzenesulfonamide (TBS-346), 4-(5-benzyl-3-(4-bromophenyl)-1*H*-1,2,4-triazol-1-yl)benzenesulfonamide (TBS-516), 4-(3-(4-bromophenyl)-5-propyl-1*H*-1,2,4-triazol-1-yl)benzenesulfonamide (TBS-546) and 4-(3-(4-bromophenyl)-5-phenethyl-1*H*-1,2,4-triazol-1-yl)benzenesulfonamide (TBS-556). Synthesis of *cis*-*cis*-4-(4-bromophenyl)-3*a*,4,5,9*b*-tetrahydro-3*H*-cyclopenta[*c*]quinoline-8-sulfonamide (4BP-TQS) and *cis*-*cis*-4-(napthalen-1-yl)-3*a*,4,5,9*b*-tetrahydro-3*H*-cyclopenta[*c*]quinoline-8-sulfonamide (TQS) has been described previously (Gill et al., 2012). NS-1738 was obtained from Tocris Bioscience.

### 2.2 Plasmids

All plasmid constructs used in this study have been described previously. These include plasmids containing cDNAs encoding human α3, α4, α7, β2 and β4 nAChR subunits in pSP64GL (Broadbent et al., 2006); rat α7 nAChR subunit in pcDNA3 (Cooper and Millar, 1997); α7/5-HT3A subunit chimera (containing the rat α7 N-terminal domain and mouse 5-HT3A transmembrane and C-terminal domain) in pcDNA3 (Cooper and Millar, 1998); human RIC-3 in pRK5 (Lansdell et al., 2005) and mouse 5-HT3A (Maricq et al., 1991) in pRK5.

### 2.3 Site-directed mutagenesis and cRNA synthesis

Site-directed mutagenesis was performed on human nAChR α7 subunit cDNA in plasmid pSP64GL using the QuikChange mutagenesis kit (Stratagene) and verified by nucleotide sequencing. Plasmid pSP64GL containing wild-type or mutated human α7 cDNA was linearized with *Bam*HI and purified with QIAQuik PCR purification kit (Qiagen). In vitro synthesis of cRNA was performed using mMessage mMachine SP6 transcription kit (Life Technologies). For consistency with previous studies, the numbering of amino acids altered by site-directed mutagenesis is based on the predicted signal sequence cleavage site in the mature α7 protein (Couturier et al., 1990).

### 2.4 Oocyte electrophysiology

Oocytes were isolated from adult female *Xenopus laevis* following procedures that have been approved by UCL's Biological Services Management Group and the UK Home Office. Oocytes were defolliculated as described previously (Young et al., 2007) by treatment with collagenase (2 mg/ml; Worthington Biochemicals, Freehold, NJ) in calcium-free Barth's solution containing 88 mM NaCl, 2.4 mM NaHCO_3_, 1 mM KCl, 0.82 mM MgSO_4_, and 15 mM Tris, pH 7.5. Heterologous expression was achieved by injection of wild-type or mutated α7 cRNA (6–12 ng) into oocyte cytoplasm. Oocytes were injected in a volume of 20–30 nl using a Drummond variable volume microinjector. After injection, oocytes were incubated at 18 °C in a calcium-containing Barth's solution (composition, as above, but with 0.77 mM CaCl_2_) supplemented with antibiotics (100 units/ml penicillin, 100 μg/ml streptomycin, 4 μg/ml kanamycin, and 50 μg/ml tetracycline). Experiments were performed on oocytes after 3–5 days of incubation. Oocytes were placed in a recording chamber and continuously perfused with a saline solution (115 mM NaCl, 2.5 mM KCl, 1.8 mM BaCl_2_, and 10 mM HEPES, pH 7.3) with a flow rate of approximately 15 ml/min. Two-electrode voltage-clamp recordings were performed (with the oocyte membrane potential held at −60 mV), as described previously (Gill et al., 2012; Young et al., 2007).

### 2.5 Radioligand binding

Human kidney tsA201 cells were cultured in Dulbecco's modified Eagle's medium (Invitrogen Life Technologies) containing 10% foetal calf serum (Sigma), penicillin (100 U/ml) and streptomycin (100 μg/ml) (Invitrogen Life Technologies). Cells were maintained in a humidified incubator containing 5% CO_2_ at 37 °C. Cells were transfected with human α7 nAChRs and human RIC-3 in 1:1 ratio using the Effectene reagent (Qiagen, Crawley, UK) according to the manufacturer's instructions. After overnight incubation in Effectene, cells were incubated at 37 °C for 24–48 h before being assayed for radioligand binding with [^3^H]-α-bungarotoxin (56 Ci/mmol; Tocris Bioscience, Bristol, UK). Radioligand binding to transiently transfected tsA201 cells was performed essentially as described previously (Lansdell and Millar 2004). Transfected cells were re-suspended in Hank's buffered saline solution (Gibco, Paisley, UK) containing 1% bovine serum albumin and incubated with [^3^H]-α-bungarotoxin for 2 h at 22 °C in a total volume of 150 μL. Non-specific binding was determined in the presence of nicotine (1 mM) and carbamylcholine (1 mM). Competition binding experiments were performed by incubating triplicate samples of transfected cells with [^3^H]-α-bungarotoxin (1 nM), together with a range of concentrations of either PAMs or methyllycaconitine (MLA). Radioligand binding was assayed by filtration onto Whatman GF/A filters (pre-soaked in 0.5% polyethylenimine), followed by rapid washing with phosphate-buffered saline (Oxoid, Basingstoke, UK) using a Brandel cell harvester. Bound radioligand was quantified by scintillation counting. Curves for equilibrium binding were fitted using GraphPad Prism (GraphPad Software, San Diego, CA, USA).

### 2.6 Statistical analysis

For pairwise comparisons, statistical significance was determined by unpaired Student's *t*-tests.

## 3 Results

### 3.1 A novel series of α7 nAChR-selective PAMs

**Fig. 1:**
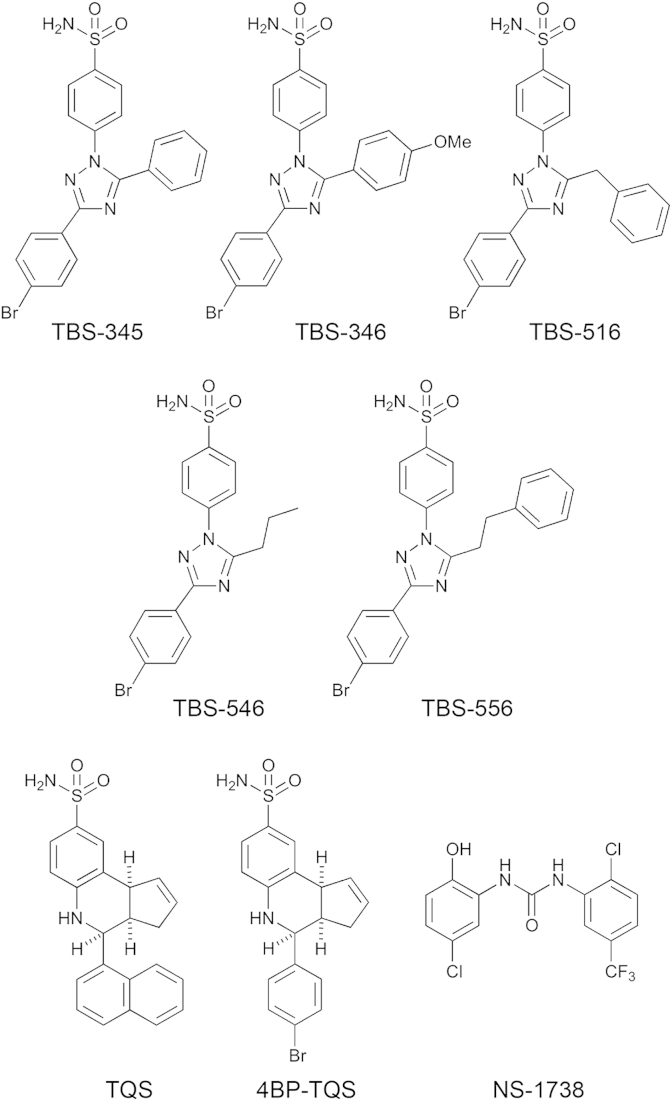
Chemical structures of the allosteric modulators examined in this study.

A novel series of compounds (Fig. 1) was constructed involving a combination of structural elements from two previously described α7-selective allosteric modulators, 4BP-TQS (Gill et al., 2011) and A867744 (Faghih et al., 2008). Both 4BP-TQS and A867744 contain an arylsulfonamide unit linked to a heterocyclic core, which has both a bromoarene and a second lipophilic group attached. Five compounds were synthesised which retained the key structural features of 4BP-TQS and A867744 but which contained a more polar triazole group as the heterocyclic core. For convenience, these compounds are referred to here collectively as ‘TBS’ compounds to reflect the fact that they all contain triazole and benzenesulfonamide groups.

**Fig. 2:**
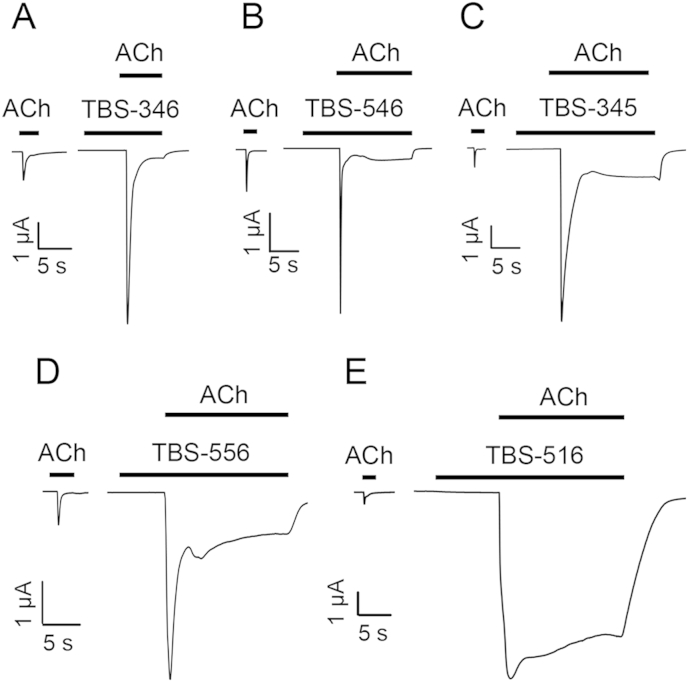
Potentiation of responses to acetylcholine on wild-type α7 nAChRs by TBS compounds. Representative traces are shown illustrating responses to acetylcholine (100 μM) together with responses from the same oocyte to acetylcholine (100 μM) after pre- and co-application of 10 μM TBS-346 (A), TBS-546 (B), TBS-345 (C), TBS-556 (D) or TBS-516 (E).

**Fig. 3:**
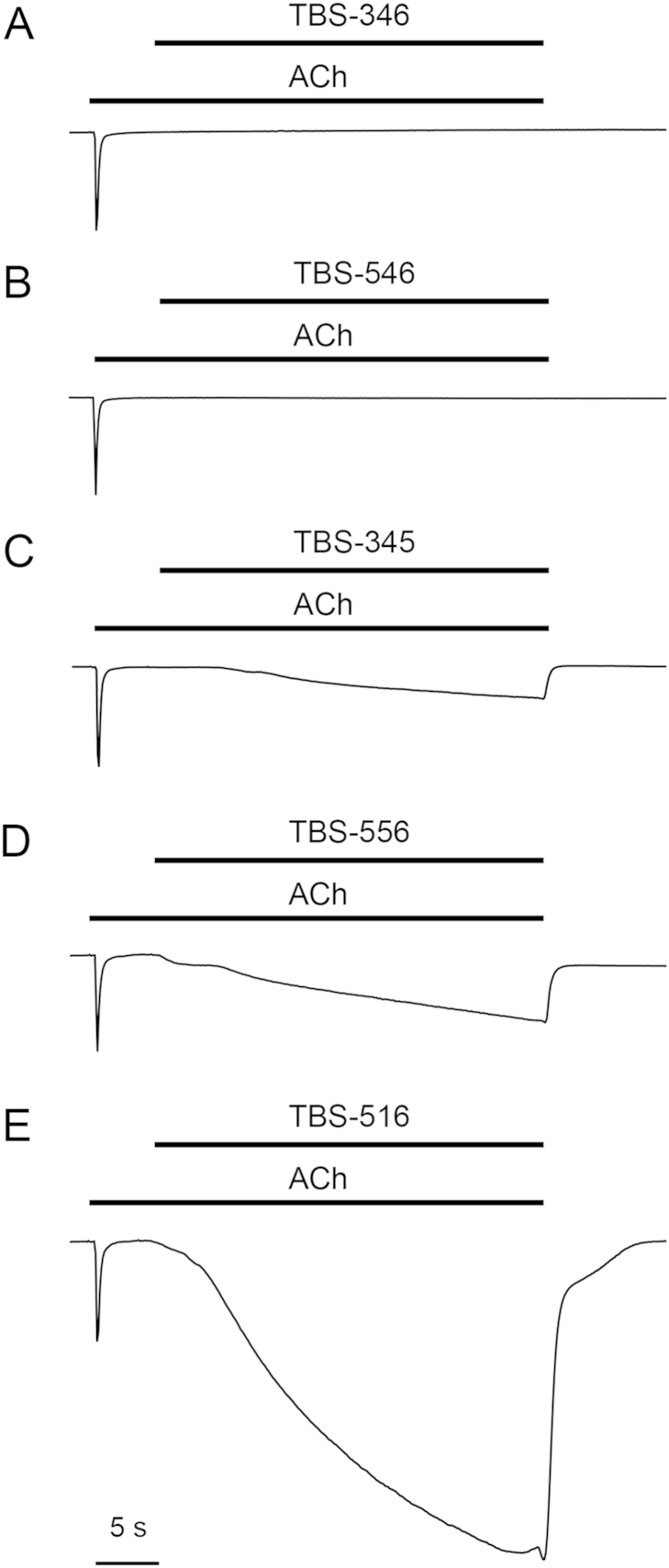
Influence of TBS compounds on the recovery of α7 nAChRs from desensitisation. Representative traces showing prolonged exposure of α7 nAChRs to acetylcholine (100 μM), causing activation, followed by rapid desensitisation. In the continued presence of acetylcholine, application of (A) TBS-346 (10 μM) and (B) TBS-546 (10 μM) does not result in reactivation of the receptor. However, application of (C) TBS-345 (10 μM), (D) TBS-556 (10 μM) and (E) TBS-516 (10 μM) results in reactivation of desensitised receptors. Traces have been scaled to their response to acetylcholine.

None of the TBS compounds examined displayed agonist activity when applied alone to human α7 nAChRs expressed in Xenopus oocytes but all of them potentiated responses evoked by acetylcholine (Fig. 2). The maximum fold potentiation by the TBS compounds of responses to an *EC*_50_ of acetylcholine (100 μM) is shown in Table 1. Notably, despite the relatively close chemical similarity between these compounds, they displayed a diverse range of effects on the rate of desensitisation of α7 nAChRs. For example, TBS-346 caused minimal changes to desensitisation, a feature that is typical of type I PAMs, whereas TBS-516 caused a dramatic slowing of desensitisation, typical of type II PAMs (Fig. 2). In addition, other compounds in this series (TBS-345, TBS-546 and TBS-556) had effects on receptor desensitisation that could be considered as being intermediate between those of classical type I and type II PAMs (Fig. 2). Another property of type II PAMs is their ability to reactivate α7 nAChRs after they have been desensitised by continuous application of an orthosteric agonist (Hurst et al., 2005), while type I PAMs lack this ability (Grønlien et al., 2007). Fig. 3 demonstrates the effect of TBS compounds on the recovery from desensitisation of α7 nAChRs after activation and desensitisation by an *EC*_50_ of acetylcholine (100 μM). TBS-346 and TBS-546 elicited no detectable responses after the receptor had been desensitised, a feature that is characteristic of type I PAMs. In contrast, TBS-345, TBS-516 and TBS-556 caused recovery from desensitisation, albeit to differing extents (Fig. 3). TBS-516 had the most profound effect, by eliciting a response that was 3.2 ± 0.9 – fold larger than the response to 100 μM acetylcholine. TBS-556 and TBS-345 elicited responses that were 57.3 ± 10.1% and 23.0 ± 2.1% of the response to 100 μM acetylcholine, respectively (Fig. 3). Where receptor reactivation was observed (when acetylcholine was co-applied with TBS-345, TBS-556 or TBS-516), no desensitisation was observed over a period 30 s (Fig. 3). The total net charge transfer, measured during a 30 s application of TBS-345, TBS-556 and TBS-516 was 12 ± 2, 27 ± 6 and 250 ± 71 fold larger than that measured during the initial application, of acetylcholine alone (Fig. 3).

**Fig. 4:**
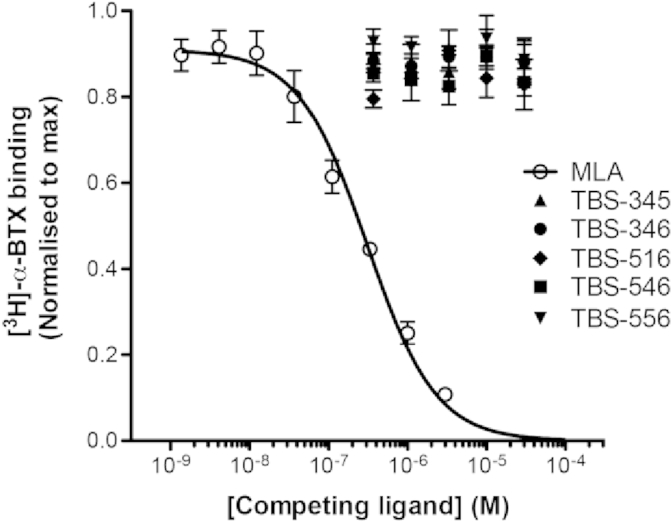
Competition radioligand binding. Equilibrium radioligand binding was performed with [^3^H]-α-bungarotoxin (1 nM) with mammalian tsA201 cells transiently transfected with human α7 nAChR subunit and with human RIC-3 cDNAs (1:1 ratio). TBS-345, TBS-346, TBS-516, TBS-546 and TBS-556 caused no significant displacement of [^3^H]-α-bungarotoxin binding, whereas MLA caused complete displacement of specific radioligand binding. Data points are means of triplicate samples (± SD) from a single experiment, and data are typical of three independent experiments.

Competition radioligand binding was performed to examine the ability of the TBS compounds to displace [^3^H]-α-bungarotoxin from the orthosteric binding site of α7 nAChRs (Fig. 4). In order to more easily obtain sufficient amounts of α7 nAChR, radioligand binding assays were performed with recombinant receptors expressed in a cultured cell line (human kidney tsA201 cells) rather than in Xenopus oocytes, which were used for electrophysiological studies. As expected, the competitive antagonist MLA fully displaced specific [^3^H]-α-bungarotoxin binding in a concentration-dependent manner (Fig. 4). In contrast, none of the TBS compounds displayed any significant displacement of [^3^H]-α-bungarotoxin binding (Fig. 4). These findings are consistent with TBS compounds acting as potentiators of α7 nAChRs via a site other than the extracellular orthosteric binding site.

All of the TBS compounds acted as potentiators of both human and rat α7 nAChRs (Table 1). In contrast, none of the TBS compounds caused potentiation of human α4β2 nAChRs, human α3β4 nAChRs or mouse 5-HT_3A_Rs. Instead, all of the compounds acted as inhibitors of this diverse group of receptors (Table 1), indicating that, to the extent that we have examined, these TBS compounds can be considered to be α7-selective PAMs. The opposing effects of the TBS compounds on α7 nAChRs and 5-HT_3A_Rs (potentiation and inhibition, respectively) prompted us to examine the effect of these compounds on an artificial subunit chimera (α7/5-HT3A) containing the extracellular/N-terminal domain of the rat α7 nAChR subunit and the transmembrane/C-terminal domain of the mouse 5-HT3A subunit. For all of the TBS compounds examined, inhibition of agonist-evoked responses was observed on the α7/5-HT3A subunit chimera (Table 1). Together, these results are consistent with these compounds interacting with the transmembrane domain, a location that has been proposed as being the site at which several other α7-selective PAMs interact with α7 nAChRs (Collins et al., 2011; Gill et al., 2011; Young et al., 2008).

### 3.2 L247T (9′) and M260L (22′) transmembrane mutations

**Fig. 5:**
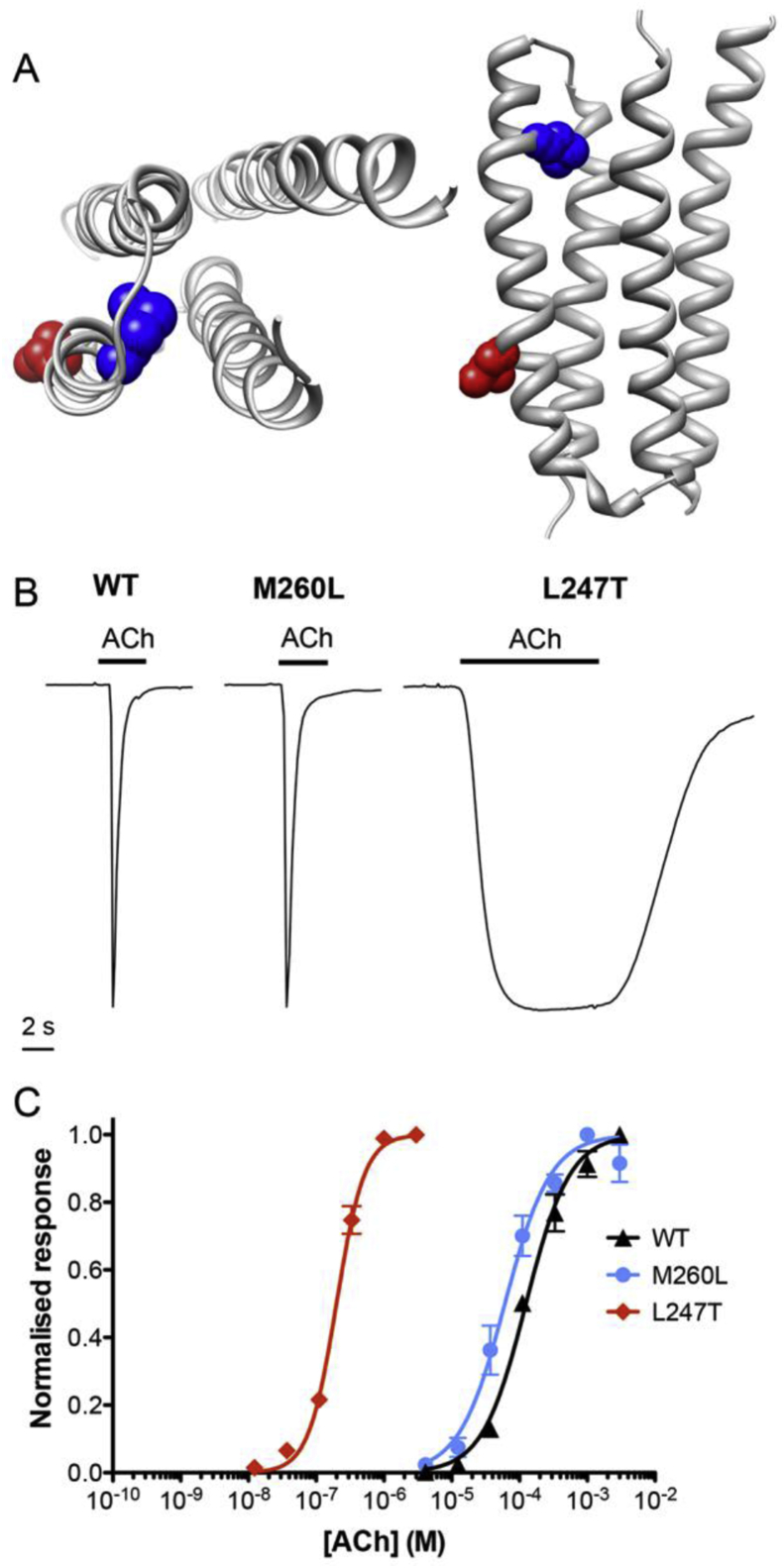
The influence of α7 nAChR mutations (L247T and M260L) on activation by acetylcholine. A) The location of L247T (9′) and M260L (22′) mutations in the α7 nAChR subunit transmembrane (TM2) domain. The transmembrane region of an α7 nAChR subunit homology model (Young et al., 2008) is shown viewed from the top (left hand image) and from the side (right hand image). The α-helical transmembrane regions are illustrated as ribbon structures with the side chains of the two mutated amino acids shown as space-filling models (L247 in red and M260 in blue). B) Representative traces are shown illustrating responses to maximal concentrations of acetylcholine on human wild-type (WT) α7 nAChRs and α7 nAChRs containing the M260L mutation (M260L) and the L247T mutation (L247T). Acetylcholine concentrations: 1 mM for WT and M260L and 10 μM for L247T. C) Acetylcholine concentration-response data are presented for wild-type α7 nAChRs (triangles) and for α7 nAChRs containing either the M260L mutation (circles) or the L247T mutation (diamonds). Data are means ± SEM of at least three independent experiments and are normalised to the respective maximum response obtained with each nAChR variant. (For interpretation of the references to colour in this figure legend, the reader is referred to the web version of this article.)

There are several examples of individual point mutations in nAChR subunits that result in dramatic effects on the pharmacological properties of receptors. For example, in agreement with previous studies (Revah et al., 1991), a single point mutation (L247T), located towards the middle of the second transmembrane (TM2) domain (at the 9′ position) of the α7 subunit (Fig. 5A) causes a dramatic slowing of receptor desensitisation and a large leftward shift (660-fold; *t*(7) = 7.1, *P* < 0.001,) of the concentration-response curve for acetylcholine (Fig. 5B and C). In contrast, another point mutation (M260L), located towards the top of TM2 (at the 22′ position) (Fig. 5A) was found to have little or no effect on the rate of receptor desensitisation after activation by acetylcholine and caused a much smaller leftward shift (2.1-fold; *t*(5) = 3.1, *P* = 0.03) in the acetylcholine concentration–response curve (Fig. 5 and Table 2).

**Fig. 6:**
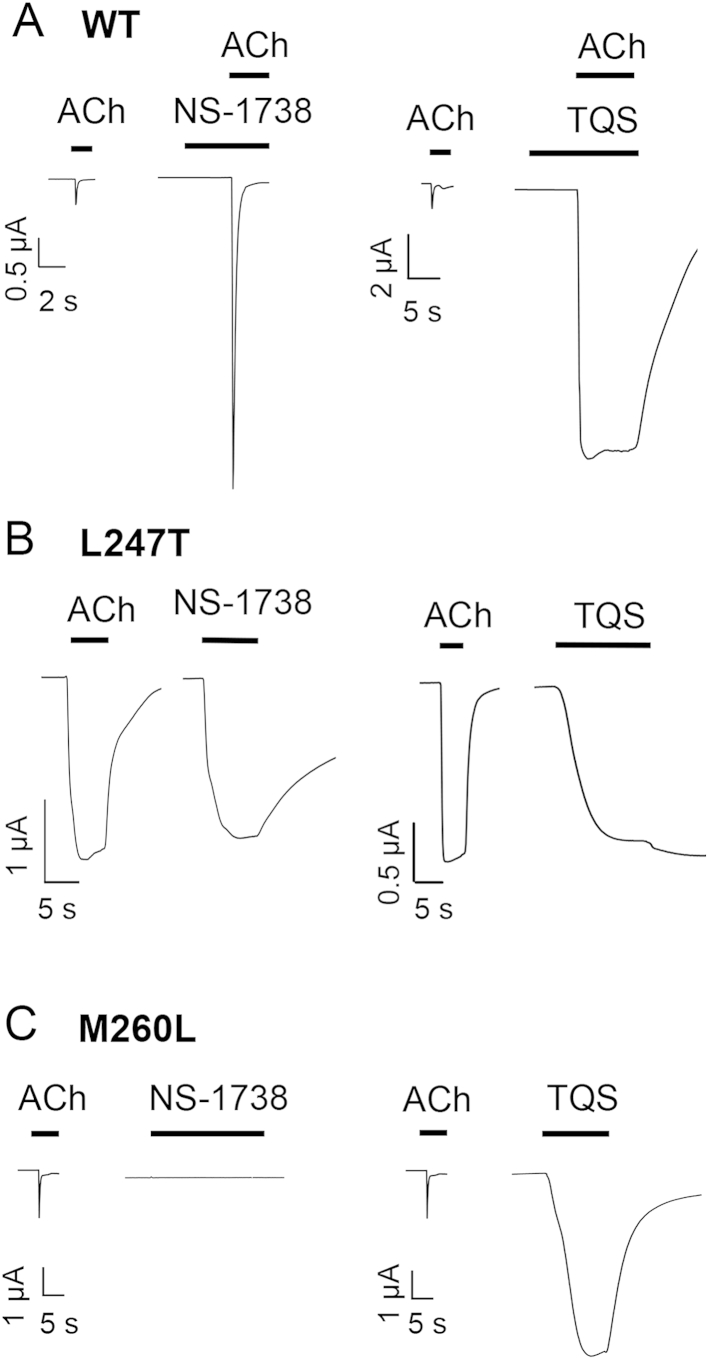
The influence of a type I (NS-1738) and a type II PAM (TQS) on wild-type and mutated (L247T or M260L) α7 nAChRs. A) Representative traces illustrating responses with wild-type α7 nAChRs to acetylcholine (100 μM) and after the pre- and co-application of either NS-1738 (10 μM; *Left pair of traces*) or TQS (30 μM; *Right*). B) Representative traces illustrating responses with mutated (L247T) α7 nAChRs to acetylcholine (10 μM) and to NS-1738 (10 μM; *Left*) or TQS (30 μM; *Right*). C) Representative traces illustrating responses with mutated (M260L) α7 nAChRs to acetylcholine (100 μM) and NS-1738 (10 μM; *Left*) or TQS (30 μM; *Right*).

We have observed further differences between wild-type and mutated (L247T and M260L) α7 nAChRs in the extent to which they are modulated by type I and type II PAMs. Initially, studies were conducted with two previously described ‘classical’ type I and type II PAMs (NS-1738 and TQS, respectively). As is illustrated, both compounds lack agonist effects on wild-type α7 nAChRs but both potentiate agonist-evoked responses on wild-type receptors (Fig. 6A). In agreement with previous studies (Grønlien et al., 2007; Timmermann et al., 2007), NS-1738 caused potentiation of agonist-evoked responses with little or no effect on desensitisation, whereas TQS results in a dramatic loss of desensitisation (Fig. 6A).

As has been reported previously (Gill et al., 2011), one of the effects of the L247T mutation is that it converts the type II PAM TQS into a potent agonist of the mutated receptor. Here we have shown that a similar effect (the conversion of a PAM into an agonist) is also observed with NS-1738, a classical type I PAM (Fig. 6B). In contrast to L247T, the M260L mutation had a selective effect on these two PAMs. As had been observed with L247T, TQS (a type II PAM) acted as a non-desensitising agonist on receptors containing the M260L mutation, whereas NS-1738 (a type I PAM) had no agonist activity on this mutated receptor (Fig. 6C). Thus, the two TM2 mutations (L247T and M260L) have differing effects on desensitisation of α7 nAChRs, when activated by acetylcholine (Fig. 5) and also differ in their ability to convert the type I PAM into an agonist (Fig. 6). However, they share an ability to convert the type II PAM into an agonist (Fig. 6).

**Fig. 7:**
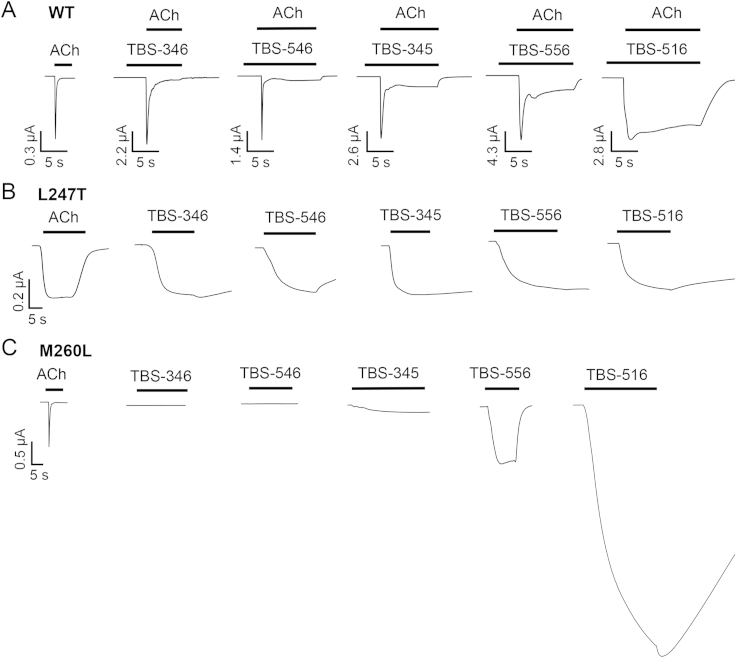
Potentiation and agonist effects of TBS compounds on wild-type and mutated α7 nAChRs. A) Representative traces are shown illustrating responses from wild-type α7 nAChRs to acetylcholine (100 μM), together with responses to acetylcholine (100 μM) after pre- and co-application of TBS compounds (10 μM). To illustrate differences in the rate of desensitsation, all responses on wild-type α7 nAChRs have been normalised to their peak response. B) Representative traces are shown illustrating agonist responses from α7 nAChRs containing the L247T (9′) mutation to either acetylcholine (10 μM) or TBS compounds (10 μM). C) Representative traces are shown illustrating agonist responses from α7 nAChRs containing the M260L (22′) mutation to either acetylcholine (100 μM) or TBS compounds (10 μM).

**Fig. 8:**
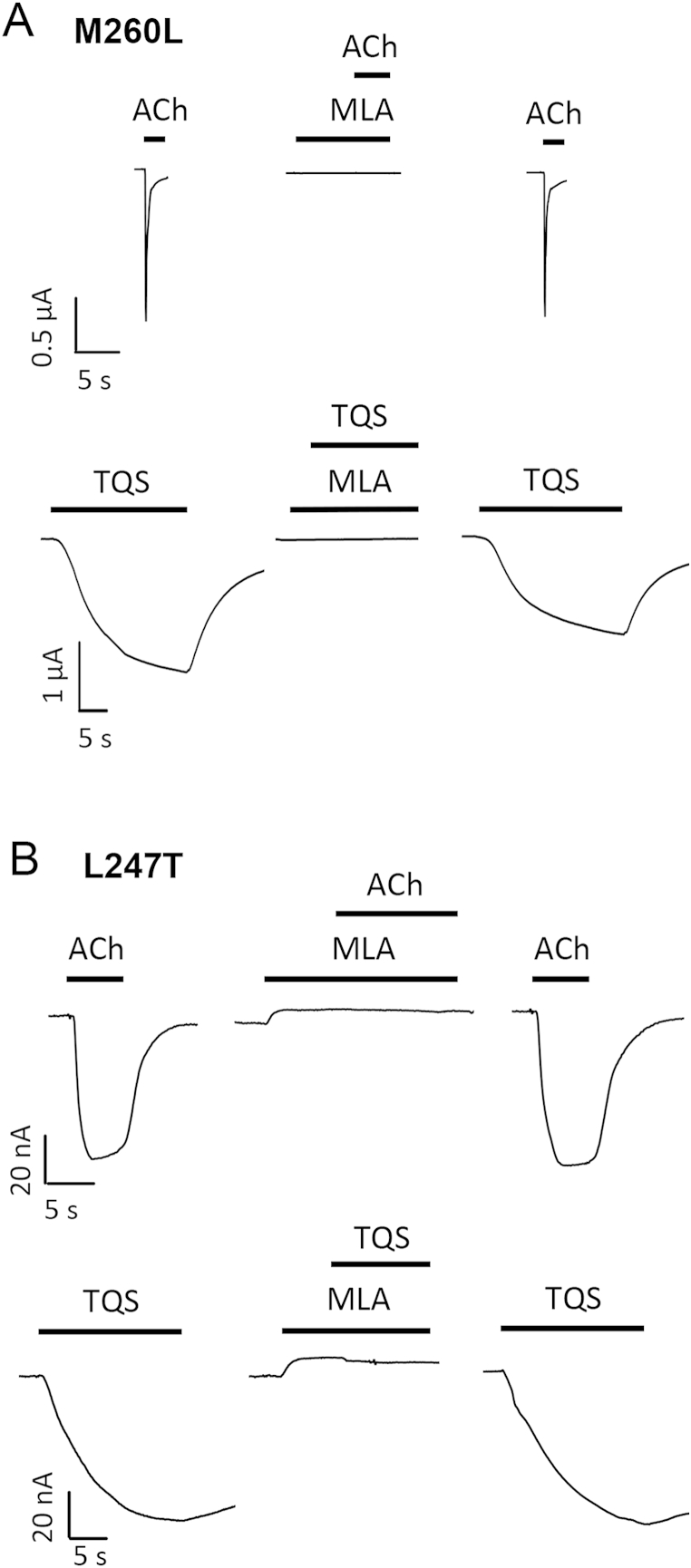
Antagonism of agonist responses to acetylcholine and TQS responses on M260L and L247T α7 nAChRs. A) Representative traces with M260L α7 nAChRs illustrating initial agonist responses with acetylcholine (1 mM) or TQS (10 μM) (left), antagonism by pre- and co-application of MLA (1 μM) (middle) and recovery in the absence of MLA (right). B) Representative traces with L247T α7 nAChRs illustrating initial agonist responses with acetylcholine (10 μM) or TQS (3 μM) (left), antagonism by pre- and co-application of MLA (1 μM) (middle) and recovery in the absence of MLA (right).

As described earlier, we have identified a series of chemically related TBS compounds that differ in their ability to alter desensitisation of α7 nAChRs (Figs. 2 and 7A). We therefore examined the influence of L247T and M260L mutations on the pharmacological properties of these compounds. The reason for doing so was to determine whether the differing effects of the L247T and M260L mutations in converting TQS but not NS-1738 into agonists reflect a consistent ability to discriminate between type I and type II PAMs. When examined on α7 nAChRs containing the L247T mutation, all five TBS compounds acted as agonists (Fig. 7B). Thus, it appears that a feature of the L247T mutation is the conversion of all PAMs examined (irrespective of their effect on desensitisation) into agonists. In contrast, with α7 nAChRs containing the M260L mutation, strong agonist activity was observed only with the two TBS compounds that had the greatest propensity to reduce desensitisation in wild-type α7 nAChRs (TBS-516 and TBS-556; Fig. 7C). Therefore, it appears that the M260L mutation has an effect on α7 nAChR structure that can discriminate between PAMs that differ in their influence upon desensitisation of wild-type α7 nAChRs. The α7-selective antagonist MLA blocked responses to acetylcholine on both L247T and M260L α7 nAChRs (Fig. 8). Similarly, MLA blocked responses with all of the allosteric modulators that act as agonists on these two mutated α7 nAChRs (representative data with TQS is illustrated in Fig. 8). This is consistent with previous reports indicating that, in addition to acting as a competitive antagonist of acetylcholine, MLA can block activation of wild-type α7 nAChRs by allosteric agonists such as 4BP-TQS via a non-competitive mechanism (Gill et al., 2011).

**Fig. 9:**
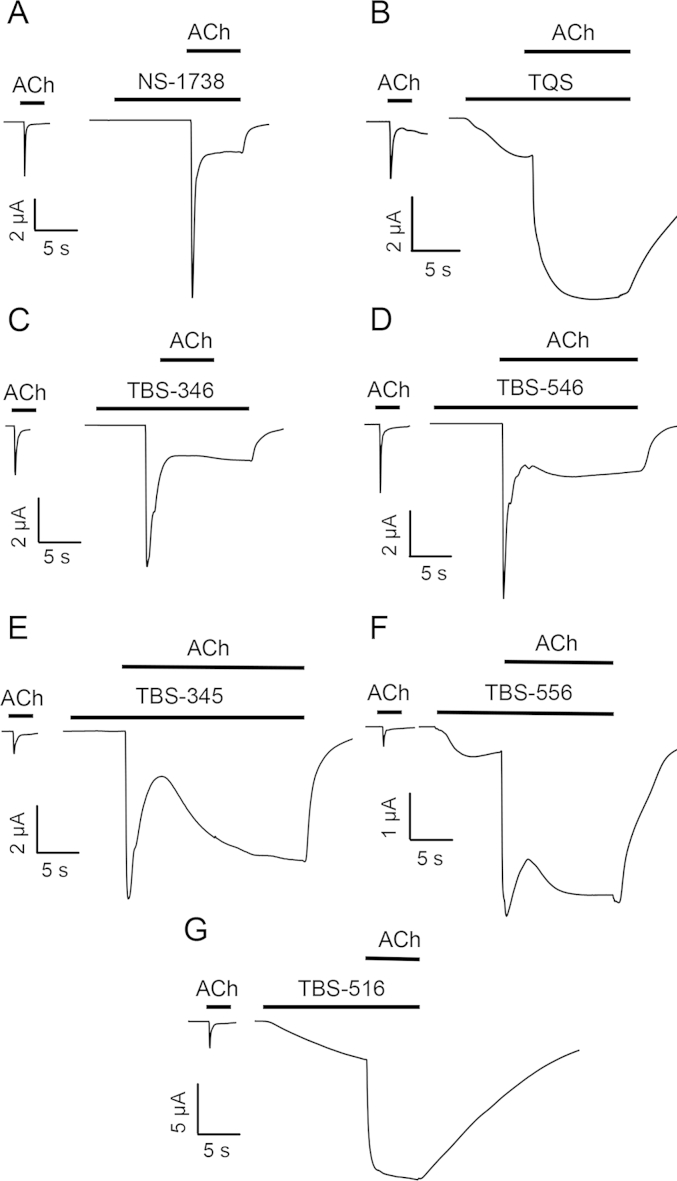
Potentiation of acetylcholine responses by allosteric modulators on M260L α7 nAChRs. Representative traces are shown illustrating responses to acetylcholine (100 μM) (left) together with responses from the same oocyte after pre- and co-application of an allosteric modulator (10 μM) (right). Representative traces are shown for NS-1738 (A), TQS (B), TBS-346 (C), TBS-546 (D), TBS-345 (E), TBS-556 (F) and TBS-516 (G).

It is notable that compounds that have no agonist effect on M260L receptors (NS-1738, TBS-346, TBS-546 and TBS-345) and also all of the compounds that are converted into agonists (TQS, TBS-556 and TBS-516) retain their ability to potentiate agonist responses when co-applied with acetylcholine (Fig. 9). The fold potentiation (determined on the basis of maximum peak response compared to acetylcholine alone) was 2.5 ± 0.3 for NS-1738, 5.8 ± 3.1 for TQS, 2.8 ± 0.6 for TBS-346, 3.6 ± 0.6 for TBS-546, 6.2 ± 1.2 for TBS-345, 6.6 ± 1.0 for TBS-556 and 8.0 ± 3.1 for TBS-516. In contrast, on L247T α7 nAChRs, upon which acetylcholine acts as a non-desensitising agonist, (Fig. 7B) the amplitude of the maximum response elicited by application of acetylcholine or by allosteric ligands was very similar (Fig. 7B and Table 2). Co-application of acetylcholine with the allosteric modulators on L247T α7 nAChRs did not potentiate or inhibit the response elicited by acetylcholine alone.

**Fig. 10:**
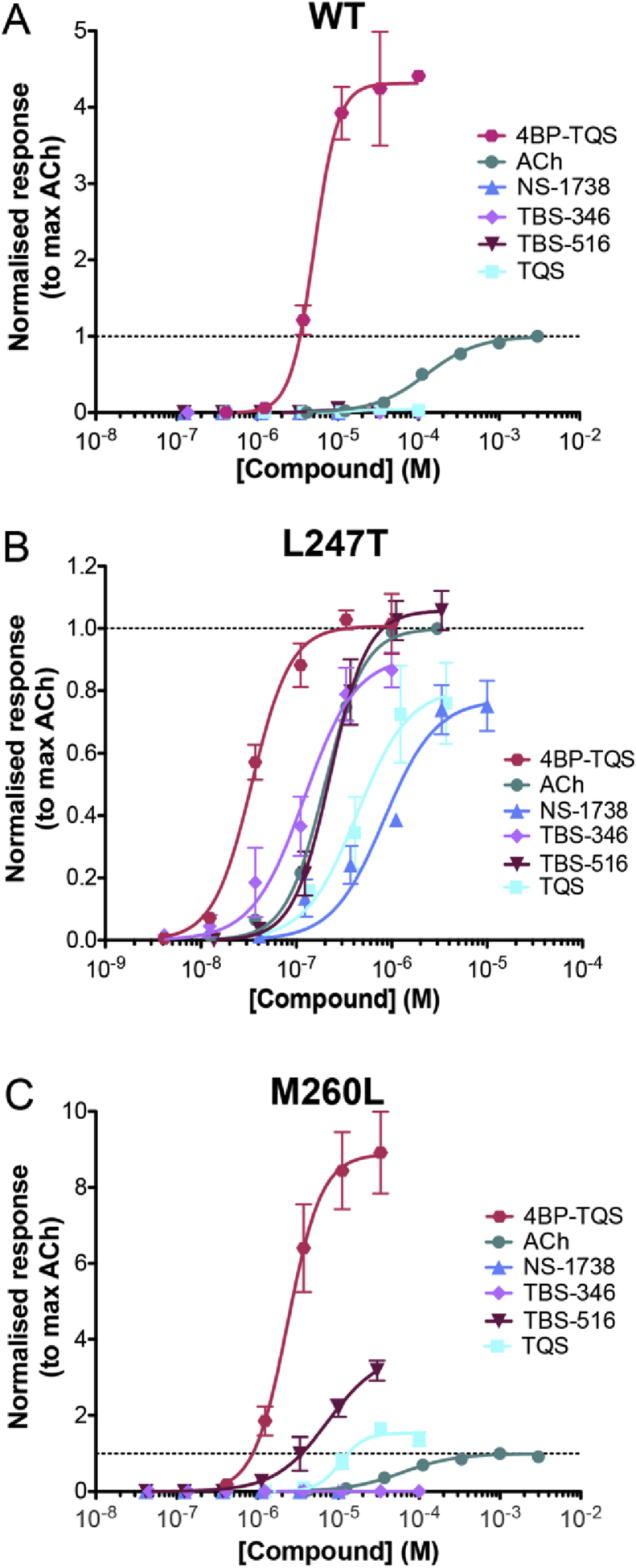
Concentration–response curves for the wild-type and mutated α7 nAChRs. Data are shown from wild-type α7 nAChRs (A), α7 nAChRs containing the L247T (9′) mutation (B) and α7 nAChRs containing the M260L (22′) mutation (C). Data are presented for a range of concentrations of acetylcholine (circles), the allosteric agonist 4BP-TQS (hexagons), the type II PAMs, TQS (squares) and TBS-516 (inverted triangles), and the type I PAMs, NS-1738 (triangles) and TBS-346 (diamonds). Data are means ± SEM of at least three independent experiments and are normalised to the maximum acetylcholine response.

For wild-type and mutated α7 nAChRs, concentration-response curves were constructed for the orthosteric agonist acetylcholine and for the allosteric agonist 4BP-TQS (Fig. 10). In addition, agonist concentration-response curves were constructed for two type I PAMs (NS-1738 and TBS-346) and for two type II PAMs (TBS-516 and TQS) (Fig. 10). In agreement with previous studies (Gill et al., 2011), the allosteric agonist 4BP-TQS generated larger maximal responses and a steeper Hill coefficient than the endogenous agonist acetylcholine on wild-type α7 nAChRs, whereas none of the PAMs tested (NS-1738, TBS-346, TBS-516 or TQS) had any detectable agonist activity on wild-type receptors (Fig. 10A and Table 2). In marked contrast, all of the compounds tested (4BP-TQS, acetylcholine, NS-1738, TBS-346, TBS-516 and TQS) generated broadly similar maximal responses and had similar Hill coefficients on α7 nAChRs containing the L247T mutation (Fig. 10B and Table 2). With α7 nAChRs containing the M260L mutation, the orthosteric and allosteric agonists (acetylcholine and 4BP-TQS, respectively) differed in their maximal responses and Hill coefficients, much as they do on wild-type α7 nAChRs (Fig. 10A and C). Also, with α7 nAChRs containing the M260L mutation, agonist responses were observed with the two type II PAMs (TBS-516 and TQS) but not with the two type I PAMs (NS-1738 and TBS-346). In addition, in contrast to the L247T mutation, maximal responses with TBS-516 and TQS (the type II PAMs) were much closer to that observed with acetylcholine than with 4BP-TQS (Fig. 10C and Table 2).

**Fig. 11:**
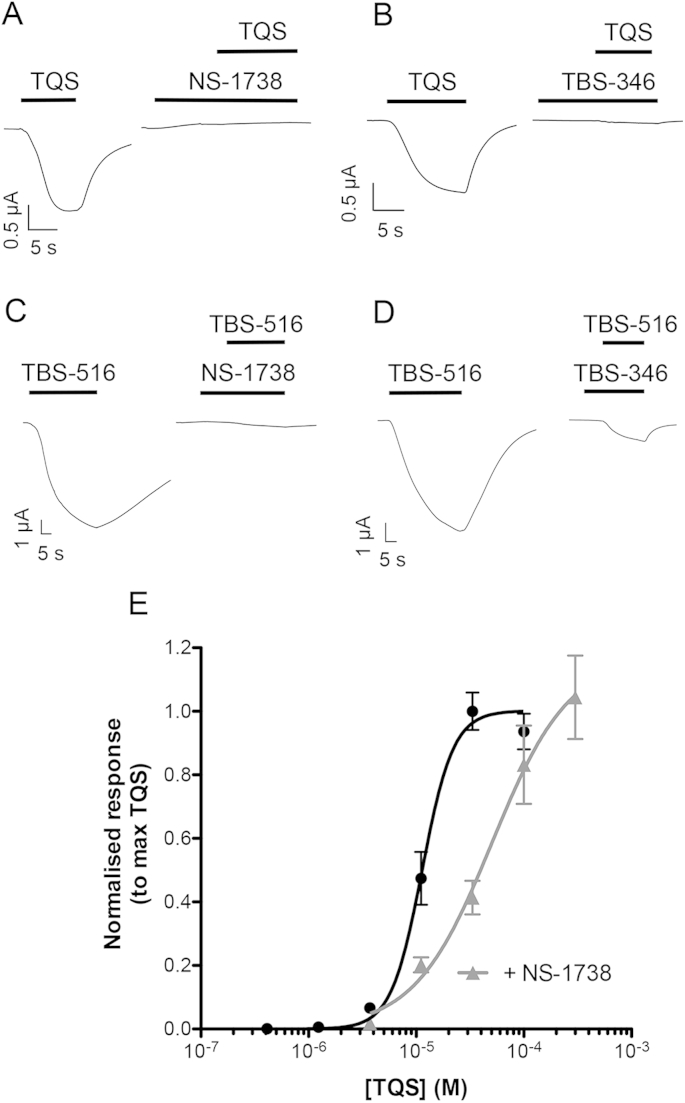
Type I PAMs block agonist activity of TQS and TBS-516 on α7 nAChRs containing the M260L mutation. Representative traces are shown, obtained by two-electrode voltage-clamp recording in oocytes expressing α7 nAChRs containing the M260L mutation, in which a type I PAM (NS-1738 or TBS-346) was pre applied for 10 s and then co-applied with a type II PAM (TQS or TBS-516) (A–D). A) NS-1738 (10 μM) completely blocked responses to TQS (10 μM). B) TBS-346 (10 μM) blocked responses to TQS (10 μM) by 95.7 ± 1.1% (n = 3). C) NS-1738 (10 μM) blocked responses to TBS-516 (10 μM) by 92.7 ± 2.3% (n = 3). D) TBS-346 (10 μM) blocked responses to TBS-516 (10 μM) by 83.4 ± 4.1% (n = 3). E) The agonist concentration-response curve for TQS on α7 nAChRs containing the M260L mutation was shifted to the right in the presence of NS-1738 (2 μM, pre-applied for 10 s and then co-applied with TQS). The antagonism by NS-1738 was surmountable at high concentrations of TQS.

Previous studies have concluded that type I and type II PAMs, despite their differing effects on desensitisation, can bind competitively at a common allosteric transmembrane site (Collins et al., 2011). Therefore, although the type I PAMs (NS-1738 and TBS-346) did not act as agonists on α7 nAChRs containing the M260L mutation (Figs. 6C and 10C) it is possible that they may block the agonist response observed with type II PAMs on this mutated receptor. When either of the type I PAMs (NS-1738 and TBS-346 at 10 μM) was pre- and co-applied with either of the type II PAMs (TQS or TBS-516 at 10 μM), an inhibition of agonist responses was observed (Fig. 11A–D). If type I PAMs are causing antagonism by binding competitively with type II PAMs on α7 nAChRs containing the M260L mutation, the antagonism would be expected to be surmountable at high concentrations of the type II PAM. We investigated this possibility by constructing an agonist concentration-response curve to TQS in the absence and the presence of a fixed concentration of NS-1738 (Fig. 11E). The *EC*_50_ for TQS was 11.5 ± 1.1 μM (n = 4) in the absence of NS-1738 and 45.4 ± 5.4 μM (n = 5) in the presence of NS-1738 (Fig. 11E). This corresponds to a significant rightward shift (4.0-fold; *t*(7) = 5.5, *P* < 0.001) of the concentration-response curve in the presence of NS-1738. However, the two curves had similar maxima (Fig. 11E), which suggests that NS-1738 is blocking responses to TQS by a competitive mechanism of action. A notable feature of the data is that the concentration–response curve is significantly less steep (*t*(7) = 3.6, *P* < 0.01) in the presence of NS-1738 (a Hill coefficient of 1.5 ± 0.2), than in the absence of NS-1738 (3.1 ± 0.4), which may be a consequence of NS-1738 acting as a potentiator of the TQS response, at low TQS concentrations, when not all sites of the receptor are occupied by TQS.

## 4 Discussion

A large number of ligands have been identified in recent years that potentiate α7 nAChRs through an allosteric mechanism of action (Arias, 2010; Bertrand and Gopalakrishnan, 2007; Faghih et al., 2008; Mazurov et al., 2011; Williams et al., 2011). In large part, this interest in α7-selective PAMs has been a consequence of the possibility that such compounds may have therapeutic use in the treatment of various neurological and psychiatric disorders (Haydar and Dunlop, 2010; Moaddel et al., 2007; Romanelli and Gualtieri, 2003; Williams et al., 2011). Traditionally, α7-selective PAMs have been characterised as either type I and type II, depending on their effect on receptor desensitisation (Bertrand and Gopalakrishnan, 2007). Type I PAMs increase peak agonist-evoked currents, without altering receptor desensitisation, whereas type II PAMs reduce the fast desensitisation of the α7 receptors. Evidence is accumulating to indicate that both type I and type II PAMs can act via a transmembrane site in α7 nAChRs (Collins et al., 2011; Young et al., 2008). In addition, allosteric agonists have been identified that appear to bind at a similar transmembrane site but, in doing so, can activate α7 nAChRs in the absence of an orthosteric agonist (Gill et al., 2013, 2012, 2011; Papke et al., 2014). Similarly, there is evidence for agonist activation, via an allosteric transmembrane site, for other pentameric ligand-gated ion channels (Amin and Weiss, 1993; Cully et al., 1996; Lansdell et al., 2015).

In the present study, five TBS compounds with close chemical similarity to one another were synthesised and were demonstrated to act as PAMs on α7 nAChRs but with range of effects on receptor desensitisation. For example, we have identified members of this series of compounds (such as TBS-346) that display effects on receptor desensitisation that are typical of type I PAMs, and others (such as TBS-516) that are more typical of type II PAMs. In addition, we have identified compounds with intermediate properties (such as TBS-546, TBS-345 and TBS-556). With several of the TBS compounds, there is evidence for two components to the rate of desensitisation of the potentiated acetylcholine-evoked response, but the proportion of the fast and slow component varied. Significantly, because of the similarity in chemical structure of these compounds, we can conclude that these differences in their ability to influence receptor desensitisation is due solely to changes in substitution at the 5-position of the triazole ring. These compounds lack PAM activity on other nAChR subtypes (such as α4β2 and α3β4) and on 5-HT_3A_Rs, indicating that they are relatively selective potentiators of α7 nAChRs. They do not displace [^3^H]-α-bungarotoxin from its orthosteric-binding site on α7 nAChRs, supporting the conclusion that these TBS compounds are allosteric modulators. In addition, we have evidence from studies of an α7/5-HT3A subunit chimera that is consistent with TBS compounds interacting with a site within the transmembrane domain.

Although previous studies have demonstrated that the L247T mutation can convert a type II PAM into an allosteric agonist (Gill et al., 2011), we have extended this finding by demonstrating that this is a feature conferred by the L247T mutation on type I PAMs, type II PAMs and also on PAMs that can be considered to have intermediate (type I/II) properties. Based on structural studies from a variety of pentameric ligand-gated ion channels, the amino acid located at a position analogous to L247 (position 9′) in the α7 nAChR is located close to the gate of the channel pore (Althoff et al., 2014; Beckstein and Sansom, 2006; Hassaine et al., 2014; Miyazawa et al., 1999). This may help to explain the profound effects that have been observed when this amino acid is mutated. It seems plausible that mutating this amino acid might disrupt the gate of the channel and may result in a receptor where more of its conformations are conducting. Indeed, a higher frequency of spontaneous openings have been reported in receptors containing the L247T mutation (Bertrand et al., 1997) as well as other changes in pharmacological properties (Bertrand et al., 1992; Palma et al., 1996; Revah et al., 1991). As has been discussed previously (Bertrand et al., 1997), it is possible that the upward deflection in current responses in response to the application of MLA to α7 L247T nAChRs (Fig. 8B) is a consequence of it blocking spontaneously open channels. In contrast to our findings with α7 nAChRs containing the L247T mutation, the M260L mutation, which is located towards the extracellular side of the TM2 domain (position 22′), has a more selective effect on PAMs. With this mutation, agonist activation was observed only with PAMs that substantially reduced the levels of desensitisation in wild-type α7 nAChRs. This effect of the M260L mutation is unlikely to be due to it preventing the binding of type I PAMs because, even though type I PAMs are not converted into agonists on the mutated receptor, they retain their PAM activity in the presence of acetylcholine. In addition, type I PAMs block agonist activation by type II PAMs in receptors containing the M260L mutation. It is thought that type I PAMs increase peak current in the presence of an agonist by facilitating the transition of the receptor from the resting to open state upon activation by the agonist without having an effect on receptor desensitisation. On the other hand, type II PAMs significantly reduce the fast desensitisation of the α7 nAChRs and may allow receptor reactivation from the desensitised state, perhaps by destabilising the desensitised state or converting the desensitised state into a new conducting state.

As is illustrated in Fig. 5A, M260 is located near the extracellular side of the TM2 domain, whereas L247 is located towards the intracellular side (at positions 22′ and 9′, respectively). It is also notable that the side chain of L247 is predicted to point towards the ion channel pore, whereas that of M260 points towards an intrasubunit cavity that has been proposed previously as a binding site for allosteric modulators of α7 nAChRs (Gill et al., 2011; Young et al., 2008). It is possible that these mutations may facilitate direct receptor activation by PAMs by altering the energy barrier for transitions between the closed, open and desensitised states of the receptor. Direct allosteric activation appears to be associated with a loss of rapid desensitisation. In the case of the M260L mutation, which has no significant effect on desensitisation, allosteric activation occurs only with type II PAMs (which themselves cause a loss of agonist-induced desensitisation). In contrast, the L247T mutation, which itself cause a loss of desensitisation, facilitates agonist activation by type I PAMs (which do not alter receptor desensitisation). The M260 residue is located towards the extracellular end of the TM2 domain in a region that has been referred to as the ‘M2-cap’ (Bafna et al., 2008). Previous studies have indicated that a stretch of 10 amino acids this region can influence allosteric modulation of an α7/5-HT3A subunit chimera (Bertrand et al., 2008). In addition, studies of this region (18′–28′) on the α1 subunit of the muscle-type nAChR, indicate that mutations in this region have large effects on gating but smaller effects on channel conductance and desensitisation (Bafna et al., 2008). However, mutating the isoleucine on the 22′ position to a leucine (which corresponds to M260 on the human α7 subunit) increased the apparent rate for entry into long-lived desensitised states by ∼10-fold (Bafna et al., 2008). It is plausible that this mutation in the corresponding residue of the α7 subunit could have an effect on the rate of receptor desensitisation, which could alter its modulation by type II PAMs.

## 5 Conclusion

In conclusion, the availability of PAMs with properties that are intermediate between those of classical type I and type II PAMs increases the pharmacological diversity of this family of allosteric modulators. In addition, evidence for mutations located at different positions in the transmembrane domain having distinct effects on allosteric modulation helps to provide a greater insight into the pharmacological diversity of these compounds. It is hoped that the ability to develop and identify compounds with differing effects on properties such as receptor desensitisation may be useful in developing useful therapeutic tools for a range of disorders.

## Figures and Tables

**Table 1:** Properties (potentiation or inhibition) of TBS compounds on nAChR and 5-HT_3A_R subtypes.

Receptor	TBS-345		TBS-346		TBS-516		TBS-546		TBS-556	
	Fold potentiation	% Inhibition	Fold potentiation	% Inhibition	Fold potentiation	% Inhibition	Fold potentiation	% Inhibition	Fold potentiation	% Inhibition
Human α7	8.8 ± 1.5	–	10.5 ± 2.5	–	11.3 ± 3.1	–	4.7 ± 1.0	–	4.7 ± 0.2	–
Human α4β2	–	20.1 ± 19.0	–	23.1 ± 5.7	–	1.7 ± 0.4	–	20.5 ± 5.9	–	3.2 ± 0.9
Human α3β4	–	53.7 ± 18.3	–	68.4 ± 3.0	–	31.1 ± 0.6	–	9.0 ± 3.5	–	16 ± 13
Rat α7	10.9 ± 0.73	–	8.7 ± 5.3	–	3.6 ± 1.0	–	1.9 ± 0.2	–	4.3 ± 0.6	–
Mouse 5-HT3A	–	18.3 ± 13.3	–	30.8 ± 15.5	–	10.4 ± 7.5	–	15.2 ± 4.3	–	10 ± 1.9
Chimera^a^	–	47.3 ± 10.1	–	13.4 ± 7.7	–	9.2 ± 12.8	–	26.4 ± 16.0	–	1.0 ± 1.0

Data are means ± SEM from at least three independent experiments.

a Chimeric subunit containing the rat nAChR α7 subunit N-terminal domain and mouse 5-HT3A subunit transmembrane and C-terminal domain.

**Table 2:** Agonist properties on wild-type and mutated α7 nAChRs.

Receptor	Agonist	EC_50_ (μM)	n_H_	I_max_^a^
Wild-type α7	Acetylcholine	132 ± 13	1.4 ± 0.2	1
	4BP-TQS	4.2 ± 0.3	5.2 ± 0.8	4.4 ± 0.3
	NS-1738	N/A	N/A	0.0 ± 0.0
	TBS-346	N/A	N/A	0.0 ± 0.0
	TBS-516	N/A	N/A	0.0 ± 0.0
	TQS	N/A	N/A	0.0 ± 0.0
α7 L247T	Acetylcholine	0.2 ± 0.01	2.1 ± 0.2	1
	4BP-TQS	0.03 ± 0.003	2.3 ± 0.2	1.0 ± 0.1
	NS-1738	0.8 ± 0.1	1.6 ± 0.2	0.8 ± 0.1
	TBS-346	0.1 ± 0.04	1.7 ± 0.3	0.9 ± 0.1
	TBS-516	0.2 ± 0.04	2.5 ± 0.3	1.1 ± 0.1
	TQS	0.4 ± 0.1	1.7 ± 0.1	0.8 ± 0.1
α7 M260L	Acetylcholine	63 ± 12	1.3 ± 0.1	1
	4BP-TQS	2.5 ± 0.4	2.3 ± 0.4	9.0 ± 1.2
	NS-1738	N/A	N/A	0.0 ± 0.0
	TBS-346	N/A	N/A	0.0 ± 0.0
	TBS-516	8.9 ± 2.5	1.8 ± 0.3	3.7 ± 0.6
	TQS	12 ± 1.1	3.1 ± 0.4	1.5 ± 0.1

Data are means ± SEM from at least three independent experiments.

a Data for maximal currents (I_max_) are normalised to the response to maximum response to acetylcholine.
