# Supplementary material for: The influence of allosteric modulators and transmembrane mutations on desensitisation and activation of α7 nicotinic acetylcholine receptors
Source: Neuropharmacology. 2015 Oct;97:75–85. doi: 10.1016/j.neuropharm.2015.05.006 (PMC4548482; doi:10.1016/j.neuropharm.2015.05.006)
Supplement: Supplementary file 1 [file mmc1.docx]

**Supporting Information**

The influence of allosteric modulators and transmembrane mutations on desensitisation and activation of α7 nicotinic acetylcholine receptors.

Anna Chatzidaki, Jarryl M. D’Oyley, JasKiran K. Gill-Thind, Tom D. Sheppard and Neil S. Millar

A novel series of potential allosteric modulators was constructed involving a combination of structural elements from 4BP-TQS^1^ and A867744^2^. Both compounds contain an arylsulfonamide unit linked to a heterocyclic core, which has both a bromoarene and a second lipophilic group attached. Five compounds were synthesised containing arylsulfonamide and bromoarene groups attached to a triazole ring but with a variety of groups attached at R (see figure).

**1-Bromo-4-(nitromethyl)benzene**^3^

AgNO_2_ (3 eq) was added to a solution of 4-bromobenzylbromide (1.5 g, 6.09 mmol) in Et_2_O (20 mL). The solution was stirred in the dark for 18 h before the solid was filtered off and the solvent removed from the filtrate under reduced pressure. The residue was purified by flash chromatography to give the nitroalkane as a white solid.

White crystals, 720 mg, 55%; Mp 54–56 °C, [55–56 °C]^3^; ν_max_ (film/cm^-1^) 1552 (N-O), 1489 (Ar), 1370 (N-O); ^1^H NMR (500 MHz, CDCl_3_) *δ* 7.58 (2H, d, *J* = 8.4, 2 × ArH), 7.34 (2H, d, *J* = 8.4, 2 × ArH), 5.39 (2H, s, CH_2_); ^13^C NMR (125MHz, CDCl_3_) *δ* 132.4, 131.7, 128.6, 124.7, 79.3; LRMS: (CI): 169 ([M-NO_2_]^+^, 100)

**(*Z*)-4-(2-((4-Bromophenyl)(nitro)methylene)hydrazinyl)benzenesulfonamide**

Prepared according to a modified literature procedure.^4^

Sulfanilamide (992 mg, 5.8 mmol) was added to stirring conc. HCl (5 mL) at RT then cooled to -5 °C. A solution of NaNO_2_ (415 mg, 6.02 mmol) in water (3 mL) was added dropwise over 5 min, then the resulting solution allowed to stir for 30 mins. Sodium acetate (4.7 g, 58 mmol) was then added in one portion and the mixture stirred vigorously. An aliquot of the solution (7.5 mL) was added to a solution of 1-Bromo-4-(nitromethyl)benzene (300 mg, 1.40 mmol) and NaOH (58 mg, 1.4 mmol) in a solution of EtOH/H_2_O (30 mL, 4:1). The solution was allowed to stir for 1 h before the precipitate was filtered and the solid was dried to give the *nitrohydrazone* as an orange solid.

Orange solid; 437 mg, 79%; Mp 165–167 °C; ν_max_ (film/cm^-1^) 3385 (N-H), 3265 (N-H), 1588 (N-O), 1492 (Ar), 1157 (S=O); ^1^H NMR (500 MHz, DMSO-d_6_) *δ* 12.03 (1H, s, NH) 7.97 (2H, d, *J* = 8.2, 2 × ArH) 7.63 (2H, d, *J* = 8.6, 2 × ArH) 7.57 (2H, d, *J* = 8.6, 2 × ArH) 7.45 (2H, d, *J* = 8.2, 2 × ArH) 4.76 (2H, s, NH_2_); ^13^C NMR (125 MHz, DMSO-d_6_) *δ* 163.7, 143.4, 142.4, 131.9, 130.1, 127.6, 127.3, 124.0, 116.9; LRMS: (ES): 497 ([M-H]^+^, 10), 369 (100); HRMS: Found (ES): [M-H]^+^ 396.9619, C_13_H_10_N_4_O_4_SBr requires 396.9606

**General Procedure for synthesis of triazoles**^5^

The nitrohydrazone (1 eq) was added to a solution of triethylamine (TEA) (1.2 eq) and primary amine (1.2 eq) in MeCN (1 M) and stirred at RT for 15 h. After this time, the solvent was removed by rotary evaporation, before addition of MeCN (2 mL), tetrapropylammonium perruthenate (TPAP) (0.2 eq) and *N*-methylmorpholine oxide (NMO) (1.5 eq). The solution was stirred for 3 h and then the solvent was removed and the residue purified by flash chromatography to give the triazole.

**4-(3-(4-Bromophenyl)-5-phenyl-1*H*-1,2,4-triazol-1-yl)benzenesulfonamide (TBS-345)**

Yellow solid, 23 mg, 50%; Mp 182–186 °C; ν_max_ (film/cm^-1^) 3315 (N-H), 2922 (C-H), 1599 (Ar), 1484 (Ar), 1342 (S=O), 1162 (S=O); ^1^H NMR (500 MHz, DMSO-d_6_) *δ* 8.06 (2H, d, *J* = 8.4, 2 × ArH), 7.92 (2H, d, *J* = 8.5, 2 × ArH), 7.73 (2H, d, *J* = 8.4, 2 × ArH), 7.67 (2H, d, *J* = 8.5, 2 × ArH), 7.53 (2H, s, NH_2_), 7.43 - 7.55 (5H, m, 5 × ArH); ^13^C NMR (125 MHz, DMSO-d_6_) *δ* 160.2, 155.1, 144.3, 140.0, 132.0, 130.5, 129.4, 128.9, 128.8, 128.0, 127.3, 127.0, 126.0, 123.1; LRMS: (CI): 454 ([M]^+^, 100), 353 (25), 170 (35), 106 (100); HRMS: Found (CI): [M]^+^ 454.010788, C_20_H_15_O_2_N_4_SBr requires 454.00991.

**4-(3-(4-Bromophenyl)-5-(4-methoxyphenyl)-1*H*-1,2,4-triazol-1-yl)benzenesulfonamide (TBS-346)**

Yellow solid, 19 mg, 40%; Mp 290–294 °C; ν_max_ (film/cm^-1^) 3400 (N-H), 1655 (Ar), 1023 (S=O); ^1^H NMR (600 MHz, DMSO-d_6_) *δ* 8.05 (2H, d, *J* = 8.3, 2 × ArH), 7.94 (2H, d, *J* = 8.5, 2 × ArH), 7.73 (2H, d, *J* = 8.3, 2 × ArH), 7.69 (2H, d, *J* = 8.5, 2 × ArH), 7.56 (2H, s, NH_2_), 7.47 (2H, d, *J* = 8.7, 2 × ArH), 7.02 (2H, d, *J* = 8.7, 2 × ArH) 3.80 (3H, s, CH_3_); ^13^C NMR (150 MHz, DMSO-d_6_) *δ* 160.8, 160.1, 155.0, 144.3, 140.3, 132.0, 130.5, 129.5, 128.1, 127.1, 126.0, 123.1, 119.4, 114.3, 55.4; LRMS: (CI): 485 ([M+H]^+^, 30), 173 (35), 85 (100); HRMS: Found (CI): [M+H]^+^ 485.026337, C_21_H_18_O_3_N_4_SBr requires 485.02830.

**4-(5-Benzyl-3-(4-bromophenyl)-1*H*-1,2,4-triazol-1-yl)benzenesulfonamide (TBS-516)**

Yellow solid, 38 mg, 45%; Mp 203–205 °C; ν_max_ (film/cm^-1^) 3365 (N-H), 3266 (N-H), 1595 (Ar), 1494 (Ar), 1328 (S=O), 1157 (S=O); ^1^H NMR (600 MHz, DMSO-d_6_) *δ* 7.99 (2H, d, *J* = 8.7, 2 × ArH), 7.98 (2H, *J* = 8.3, 2 × ArH), 7.83 (2H, d, *J* = 8.7, 2 × ArH), 7.70 (2H, d, *J* = 8.3, 2 × ArH), 7.57 (2H, s, NH_2_), 7.28 (2H, t, *J* = 7.3, 2 × ArH), 7.22 (1H, t, *J* = 7.3, ArH), 7.17 (2H, d, *J* = 7.3, 2 × ArH ), 4.34 (2H, s, CH_2_); ^13^C NMR (150 MHz, DMSO-d_6_) *δ* 159.9, 156.1, 144.3, 139.3, 135.8, 132.0, 129.5, 128.7, 128.6, 128.0, 127.1, 126.9, 125.4, 123.0, 32.1; LRMS: (CI): 469 ([M]^+^, 100); HRMS: Found (CI): [M]^+^ 469.030795, C_21_H_17_O_2_N_4_SBr requires 469.03338

**4-(3-(4-Bromophenyl)-5-propyl-1*H*-1,2,4-triazol-1-yl)benzenesulfonamide (TBS-546)**

Yellow solid, 24 mg, 29%; Mp 204–208 °C; ν_max_ (film/cm^-1^) 3321 (N-H), 3073 (C-H), 1597 (Ar), 1497 (Ar), 1340 (S=O), 1161 (S=O); ^1^H NMR (600 MHz, DMSO-d_6_) *δ* 8.02 (2H, d, *J* = 8.7, 2 × ArH), 7.99 (2H, d, *J* = 8.5, 2 × ArH), 7.87 (2H, d, *J* = 8.7, 2 × ArH), 7.70 (2H, d, *J* = 8.5, 2 × ArH), 7.58 (2H, s, NH_2_), 2.87 (2H, t, *J* = 7.5, C(N)CH_2_), 1.75 (2H, sxt, *J* = 7.5, C*H*_2_CH_3_), 0.92 (3H, t, *J* = 7.5, CH_3_); ^13^C NMR (150 MHz, DMSO-d_6_) *δ* 159.7, 157.4, 144.1, 139.4, 131.9, 129.7, 128.0, 127.1, 125.3, 122.9, 28.0, 20.3, 13.6; LRMS: (CI): 421 ([M+H]^+^, 100), 343 (10); HRMS: Found (CI): [M+H]^+^ 421.032564, C_17_H_18_O_2_N_4_SBr requires 421.03338

**4-(3-(4-Bromophenyl)-5-phenethyl-1*H*-1,2,4-triazol-1-yl)benzenesulfonamide (TBS-556)**

Orange powder, 32 mg, 33%; Mp 184–186 °C; ν_max_ (film/cm^-1^) 3282 (N-H), 3015 (C-H), 1495 (Ar), 1332 (S=O), 1159 (S=O); ^1^H NMR (600 MHz, DMSO-d_6_) *δ* 8.02 (2H, d, *J* = 8.7, 2 × ArH), 7.98 (2H, d, *J* = 8.7, 2 × ArH), 7.74 (2H, d, *J* = 8.5, 2 × ArH), 7.72 (2H, d, *J* = 8.5, 2 × ArH), 7.56 (2H, s, NH_2_), 7.23 - 7.27 (2H, m, 2 × ArH), 7.16 - 7.20 (3H, m, 3 × ArH), 3.19 (2H, t, *J* = 7.8, C(N)CH_2_), 3.09 (2H, t, *J* = 7.8, PhCH_2_); ^13^C NMR (150 MHz, DMSO-d_6_) *δ* 159.7, 156.8, 144.1, 140.3, 139.3, 132.0, 129.6, 128.44, 128.38, 128.0, 127.1, 126.3, 125.2, 122.9, 32.7, 28.2; LRMS: (CI): 483 ([M+H]^+^, 100), 111 (55); HRMS: Found (CI): [M+H]^+^ 483.047112, C_22_H_20_O_2_N_4_SBr requires 483.04903

**References**

(1) Gill, J. K.; Dhankher, P.; Sheppard, T. D.; Sher, E.; Millar, N. S. *Mol. Pharmacol.* **2012**, *81*, 710–718.

(2) Faghih, R.; Gopalakrishnan, S. M.; Gronlien, J. H.; Malysz, J.; Briggs, C. A.; Wetterstrand, C.; Ween, H.; Curtis, M. P.; Sarris, K. A.; Gfesser, G. A.; El-kouhen, R.; Robb, H. M.; Radek, R. J.; Marsh, K. C.; Bunnelle, W. H.; Gopalakrishnan, M. *In Vitro* **2009**, 3377–3384.

(3) Kornblum, N.; Brown, R. A. *J. Am. Chem. Soc.* **1964**, *86*, 2681–2687.

(4) Avramenko, G. V; Bezulglaya, Z. V; Stepanov, B. I.; Sheban, G. V. *J. Gen. Chem. USSR* **1990**, *60*, 364.

(5) El Kaim, L.; Gizzi, M.; Grimaud, L. *Synlett* **2010**, 1771–1774.
